# Gleanings in Science and Medicine

**Published:** 1893-06-10

**Authors:** 


### Gleanings in Science and Medicine.

The reason why we have been endowed by Nature with two ears is briefly explained in the pages of a weekly contemporary. It is impossible, affirms the writer of this article, for anyone properly to locate sound without the balancing evidence of two organs of hearing, because, as regards the hearing of the individual, sound moves in direct lines from the cause of disturbance to the ear. Persons, therefore, who have lost the hearing of one organ, have lost thereby the essential guide to comparison of sound, which is dependent on the evidence of the two.

Dr. Schofield's recent lecture at the National Health Society concerning the approaching cholera epidemic, whilst it contained nothing very new, is worthy the notice of all, as being a lesson in sound common sense. It is not certain that we can escape from the infection of any ravaging disease, but we can all prepare ourselves against it. "The condition of a person when attacked, resembles the condition of a town when besieged by a hostile army—it depends upon the supplies within the walls" the lecturer observed. Thus it plainly is the duty of all within the range of the epidemic to keep up the best possible strength, for it is on this reserve strength, that the actual virulence of the attack is dependent. This is a timely warning, and one we should all take to heart, and prepare each our own individual selves against the coming of the evil day.

Students of epidemiology have lately had a wider field of speculation opened out for them by Dr. Charrin's investigations on disease germs, the results of which he has published in the *Academie des Sciences*. These pathogenic microbes are equally injurious, he asserts, in the vegetable as in the animal kingdom, though the actual harm they effect in the case of the former is less pernicious on account of the greater resistance offered to disease germs entering into the circulatory system of plants than through the ordinary epidermis of animals. But though the actual structure of plants gives less encouragement to the entrance of these pathogenic germs, yet disease thus disseminated is of an equally injurious nature in both kingdoms, animal and vegetable.

A pamphlet entitled "Free Human Flying, as the Preliminary Condition of Dynamic Æronautics" has lately attracted some considerable attention in Bavaria, of which country, Herr Koch, the author, is a native. The theory of this new flying apparatus is soon to assume a practical form, and to enable it to be further perfected and tested the Ministers of the Interior and Education of Bavaria have granted Herr Koch a sufficient sum of money to carry out his invention forthwith. The results will be awaited with a certain interest, for though this is by no means the first flying machine of which we have heard, yet a possibility of success appears to be attached to it, considering that the Government has afforded financial support and encouragement towards its perfection.

